# Mpox and Lessons Learned in the Light of the Recent Outbreak: A Narrative Review

**DOI:** 10.3390/v16101620

**Published:** 2024-10-16

**Authors:** Konstantinos Protopapas, Dimitra Dimopoulou, Nikolaos Kalesis, Karolina Akinosoglou, Charalampos D. Moschopoulos

**Affiliations:** 1 Fourth Department of Internal Medicine, Attikon University Hospital, School of Medicine, National and Kapodistrian University of Athens, 12462 Athens, Greece; kprotopapas@hotmail.com (K.P.); chamos@med.uoa.gr (C.D.M.); 2 Second Department of Pediatrics, “Aghia Sophia” Children’s Hospital, 11527 Athens, Greece; dimi_med@hotmail.com; 3 Dermatology Department, General Hospital of Nikaia-Piraeus ‘Agios Panteleimon’, General Hospital of West Attica ‘Agia Varvara’, 12351 Athens, Greece; nickkalesis@gmail.com; 4 Department of Medicine, University of Patras, 26504 Rio, Greece

**Keywords:** monkeypox virus, mpox, zoonotic diseases, outbreak, vaccination, global response

## Abstract

According to the WHO, more than 90,000 cases of mpox have been reported since the 2022 worldwide outbreak, which resulted in 167 deaths, while a new outbreak in Africa since 2023 has resulted in over 18,000 cases and 617 deaths. Mpox is a zoonosis caused by the monkeypox virus, a double-stranded DNA virus belonging to the Orthopoxvirus genus, which causes smallpox-like illness. Until 2022, cases were predominately located in West and Central Africa, with only sporadic cases and outbreaks reported in other parts of the world. During the 2022 outbreak, the primary mode of transmission was sexual contact among men who have sex with men. The changing epidemiology of mpox resulted in new disease phenotypes and populations at risk, disproportionally affecting people who live with HIV. Commonly presenting as a mild, self-limiting illness, mpox can cause severe and protracted disease in people with HIV with a CD4 count < 200 cell/mm^3^. The global emergence of mpox that followed and intersected with COVID-19 mobilized the scientific community and healthcare stakeholders to provide accurate diagnostics, preventive vaccines and treatment to those most affected. Despite existing gaps, this rapid response helped to contain the outbreak, but challenges remain as new variants emerge. Preparedness and readiness to respond to the next outbreak is crucial in order to minimize the impact to the most vulnerable.

## 1. Introduction

The worldwide outbreak of mpox (previously known as monkeypox) in 2022 brought this previously neglected disease into the spotlight [1]. Endemic in West and Central Africa for decades, mpox emerged, for reasons not yet quite understood, in the aftermath of the COVID-19 pandemic, but it also intercepted with the HIV epidemic, disproportionately affecting people living with HIV (PLWH) and men who have sex with men (MSM) [2]. The reflexes of the scientific community and health policymakers were immediate as they swiftly mobilized resources and implemented measures to address the situation [3]. The preceding COVID-19 pandemic had laid the groundwork for the rapid recognition and surveillance of the mpox epidemic as well as vaccine production and delivery, public communication and the implementation of preventive strategies to contain the disease. The already-established networks of sexual health/HIV services and community stakeholders took on the task of informing the populations most at risk and implementing prevention, testing and treatment protocols [4,5,6]. This review outlines the origins and virology of the monkeypox virus (MPXV) as well as its clinical presentation, diagnosis and treatment. It also lays out the available preventive measures, with a focus on vaccination, and discusses the preparedness of global and community leaders to effectively respond to this outbreak as the world anticipates the next one.

## 2. Taxonomy and Virology

MPXV is an enveloped double-stranded DNA virus belonging to the Poxviridae family of the genus *Orthopoxvirus* and is closely related to vaccinia virus, the cause of smallpox [7]. The name of the virus originates from its discovery in 1958 in Copenhagen in laboratory cynomolgus monkeys (*Macaca fascicularis*) presenting smallpox-like disease [8]. Although the primary host of the virus is still elusive, various African rodents have been reported to be potential reservoirs, whereas apes and monkeys are considered to be intermediate hosts [9,10]. After the 2022 outbreak, a nomenclature change was suggested to destigmatize the disease, resulting in the adoption of the name mpox for the disease caused by MPXV by the World Health Organization (WHO). A consensus was also reached to rename the two viral clades, previously known as Congo Basin and West African, to Clades I and II, respectively. Furthermore, two subclades of Clade II were recognized, with Clade IIb referring to the virus that caused the 2022 outbreak [10,11,12]. More recently, an offset of Clade I was identified, named Clade Ib, that caused an outbreak with sustained human-to-human transmission in the Democratic Republic of the Congo (DRC) [13].

**Figure 1:**
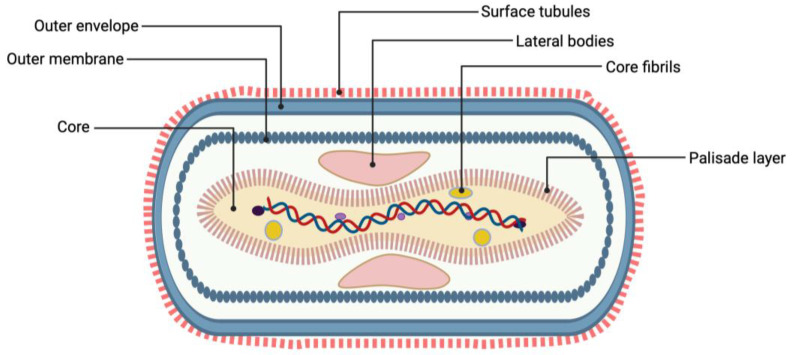
Structure of monkeypox virus (created using https://www.biorender.com (accessed on 7 July 2024)).

Poxviruses are characterized by their cytoplasmatic replication, in contrast to other DNA viruses whose reproduction takes place inside the cell nucleus [14]. Moreover, they are among the largest viruses, with MPXV reaching a length of 220–450 nm and having a brick-like shape [15]. The core of the mature virion contains 197 kb long linear dsDNA and core fibrils, and it is surrounded by the core membrane and a palisade layer that gives the core its characteristic biconcave shape. Two lateral bodies are located between the core and the outer membrane, while spontaneously released virions are also surrounded by a lipoprotein envelope (Figure 1) [15,16,17]. Due to their large size, poxviruses are easily detected by host immune defenses and have a more prolonged replication cycle in comparison with smaller viruses. Therefore, they employ elaborate strategies to survive in the host cell, producing molecules that disrupt the host’s defenses, both within the cell and extracellularly [18,19]. Intracellular virulence factors include virotransducer and virostealth proteins, which act by dampening the cell’s response to infection and by reducing the detection of the infected cell by the immune system, respectively [20,21]. On the other hand, viromimic proteins act in the extracellular space and include viroreceptors and virokines. Viroreceptors bind and virokines mimic chemokines and cytokines secreted by the host, consequently evading their antiviral action and promoting viral survival and replication [19,22]. Despite the abundance of these virulence factors in all poxviruses, each virus exhibits its own set of immune-evasion tactics, resulting in pathogenic differences, even within the same species [23]. For example, Clade I MPXV expresses a complement-binding protein that interferes with complement activation, which is absent from Clade II MPXV [23,24]. This may partly explain the higher fatality rate of mpox in Central Africa (10–17%), where Clade I is prevalent, and the relatively more benign disease during the 2022 epidemic caused by the Clade IIb variant [25,26]. Moreover, facilitated human-to-human transmission of both the Clade IIb and Ib MPXV has been proposed to result from noncanonical viral evolution driven by the accumulation of characteristic mutations associated with the action of the apolipoprotein B messenger RNA editing enzyme catalytic polypeptide 3 (APOBEC3) [13,27]. APOBEC3 enzymes belong to the cytidine deaminase family (they deaminate cytosine to uracil), introduce G → A mutations to the complementary DNA and demonstrate antiviral properties [28]. Experimentally, human APOBEC3F has been found to cause hypermutations in the MPXV genome, which may have driven the viral evolution that has been seen to contribute to the recent epidemics [27,29,30].

## 3. Origin and Epidemiology

### 3.1. West and Central Africa

After its first appearance in 1958, MPXV outbreaks in imported captive primates were recorded in the US and the Netherlands for the next 10 years, yet no human case was recorded [31]. In 1970, the first case of mpox in humans was reported in a 9-month-old child from the DRC, who presented with smallpox-like disease [32,33]. Since then, several West and Central African countries have reported sporadic cases and self-contained outbreaks related to animal contact, with limited human-to-human transmission. In the period 1996–1996, the DRC recorded a more serious outbreak with more sustained human-to-human transmission [34]. As the universal variola vaccination, which was 85% effective in preventing mpox, was halted after the success of the Smallpox Eradication Programme in 1980, population immunity gradually waned [35]. The first outbreak outside Africa was recorded in the USA in 2003 and originated from prairie dogs, which in turn had been infected by imported rodents from Ghana. During this outbreak, MPXV Clade II caused 72 confirmed or suspected cases, with no proven human-to-human transmission or fatalities being reported [36,37].

In the meantime, mpox remained endemic in the DRC, with cases also recorded in 9 other countries in West and Central Africa [38]. Surveillance of the disease showed a steadily increased incidence between 1980 and 2005 [39], continuing through 2013 [30]. In early 2022, a systematic review showed that published cases almost doubled between 2000 and 2009 and 2009 and 2019, with the majority of cases reportedly being caused by Clade I [40]. In 2017, after 40 years of no reported cases, Nigeria reported its first outbreak of Clade II mpox, which was the source of the first exported human cases outside Africa that were recorded in the United Kingdom, Israel and Singapore [41]. This outbreak was characterized by sustained human-to-human transmission, while the epidemiological data suggested sexual contact to be among the possible modes of transmission [42]. This outbreak was caused by the Clade IIb virus that eventually gave rise to the MPXV lineage B.1, the cause of the 2022 global outbreak [43].

### 3.2. 2022 Global Outbreak

In early May 2022, the WHO reported the first cases of mpox in the United Kingdom that were not related to travel to endemic countries, raising concern for community spread, and by mid-May 2022, cases were reported in 12 countries [44]. Gradually, more countries started to report cases in all six WHO regions. On 23 July 2022, the WHO Director-General declared this outbreak as a Public Health Emergency of International Concern (PHEIC) [45]. As of 7 February 2024, nearly 92,000 cases of mpox have been recorded in more than 100 countries not endemic for MPXV, resulting in 156 deaths [46]. The worldwide epidemic disproportionately affected MSM, while intimate sexual contact was the main route of transmission [47]. The epidemic reached its peak in August 2022 and, despite cases being on the decline since then, low levels of transmission are still reported [48]. This outbreak, caused by a new MPXV lineage, marked a significant change in mpox epidemiology, risk factors and clinical presentation and raised awareness of a previously neglected disease. The PHEIC was declared to be over in May 2023 after there had been a sustained decline in global cases [49].

### 3.3. 2023–2024 Outbreak

As early as September 2023, an epidemic of a new variant of Clade I MPXV, called Clade 1b, began in Central Africa [50]. The Africa Centres for Disease Control and Prevention (ACDC) reported an increase of approximately 160% from the previous year [51]. During 2024, over 21,000 mpox cases due to MPXV Clade I and Clade II have been reported from 13 African Union Member States, including over 3000 confirmed cases, in Burundi, Cameroon, Central African Republic, Republic of the Congo (hereafter referred to as Congo), Côte d’Ivoire, DRC, Ghana, Liberia, Kenya, Nigeria, Rwanda, South Africa and Uganda [52]. In DRC, most cases and deaths reported were among those < 15 years of age, representing 66% of the total cases and 82% of the total deaths. Males accounted for 73% of the cases in DRC. In Congo, based on information provided by the Africa CDC, most confirmed cases (56%) were children < 15 years of age and 58% were male; similarly, in the Central African Republic, 43% of the confirmed cases were < 15 years of age and 62% were male [53].

As of August 2024, 18,737 suspected cases and 617 deaths (case fatality rate: 2.57%) have been reported in 13 African countries, nearly all in the DRC [52]. This rate is significantly higher than the previously reported fatality rate of less than 0.1% recorded during the 2022–2023 mpox outbreak [47]. However, the size of these outbreaks could be much larger than reported due to under-ascertainment and under-reporting in those areas [54]. Consequently, the true number of infections is likely to be higher than the reported figures, while the case fatality rate may have been overestimated. Moreover, it is reasonable to assume that community transmission is occurring in several African countries, considering the wide geographic distribution of cases and the diverse age groups involved.

As a result, on 14 August 2024, the World Health Organization declared the epidemic to be a second Public Health Emergency of International Concern (PHEIC) [50]. On 15 August 2024, Sweden reported the first imported case of mpox due to MPXV Clade Ib in EU/EEA countries [55]. Hence, on 16 August, the European Centre for Disease Prevention and Control (ECDC) adjusted the risk level of Clade I for the European population, raising it from “very low” to “low” in response to the anticipated rise in imported cases across the region. Nonetheless, the ECDC noted that the risk depended on type of contact and the population immunity setting. Despite this, the agency stressed that the probability of the ongoing transmission of the virus strain within Europe remained minimal [56].

## 4. Risk Factors

Understanding the risk factors associated with mpox transmission and spread is important to implement effective prevention and infection control strategies. Zoonotic transmission occurs through direct contact with bodily fluids, the mucosal and cutaneous lesions of infected animals such as rodents and primates or the consumption of undercooked meat from infected animals [57]. This is a significant risk factor, particularly in regions where interactions between humans and wildlife is common, including in rural areas with dense forests or regions with tropical rainforests such as Central and West Africa [57]. Despite human-to-animal transmission being uncommon, a few cases of human-to-dog transmission have been reported [58,59]. Although zoonotic transmission is the primary mode of infection, human-to-human transmission may prevail during outbreaks, involving the inhalation of respiratory droplets or direct contact with the lesions or bodily fluids of a patient [57]. Factors such as overcrowding, poor sanitation and inadequate healthcare infrastructure may facilitate the spread of mpox [57,60]. Nosocomial outbreaks and the transmission of MPXV during laboratory research as well as vertical transmission through the placenta have also been reported [61,62]. Socioeconomic disparities, including communities with limited access to healthcare, education and resources and cultural practices, including the hunting, handling or consumption of wildlife, are additional risk factors for mpox [57,60].

Primary zoonotic mpox is more common in men, as they are more likely to be hunters, and in younger individuals, as they are more likely to eat undercooked infected animals [63]. In the 2022 outbreak, however, among the cases with known data on sexual behavior, 85.5% identified as MSM; among the cases who reported a type of transmission, 83.5% reported a sexual encounter [48]. Sexual transmission has been suggested because virus DNA has been detected in seminal fluid [64]. The R(t) (reproduction number) of mpox during the current outbreak in Italy was 2.43 among MSM, showing that the virus has the potential to cause epidemics in this population [65]. People unvaccinated against smallpox are at an increased risk of infection, while pediatric patients or young and immunocompromised adults such as PLWH are at a higher risk of severe disease [66,67].

### Mpox in People Living with HIV

A recent systematic review and meta-analysis showed that the pooled prevalence of HIV infection among patients diagnosed with mpox was 41% [68]. More specifically, studies from Europe and North America reported high prevalences of HIV co-infection of about 41% and 52%, respectively, while studies from Nigeria in Africa reported a relatively low prevalence of 21% [68]. During the 2017/2018 Nigerian mpox outbreak, 22.5% of hospitalized patients with mpox were PLWH. In the 2022 global mpox outbreak, 52% of the confirmed mpox cases were PLWH, predominantly sexual and gender minority groups, indicating that sexual activity was underestimated as a major risk of transmission before the 2022 outbreak [69]. Living with HIV, receiving pre-exposure prophylaxis (PrEP) or reporting ≥ 20 sexual partners in the past 12 months as well as sex in sex venues/parties in the past 2 months were found to be independent risk factors for mpox diagnosis [70]. It has been reported that PLWH with mpox have a higher frequency of perioral lesions and pharyngitis as well as a higher number of sexually transmitted infections [67,71].

Individuals with advanced or uncontrolled HIV infection are at a higher risk of severe mpox [66,72,73]. The combination of HIV-induced immunosuppression and the virulence of the monkeypox virus may result in increased viral replication, prolonged disease and a higher risk of complications [73]. Severe complications and systemic manifestations were more common in people with a CD4 cell count < 100 cells/μL than in those with > 300 cells/μL, while death was observed in people with CD4 counts of < 200 cells/μL and with a high HIV viral load [2]. Mpox severity was also related to poor HIV continuum of care outcomes and low CD4^+^ cell counts, while increased mortality was reported in PLWH with CD4^+^ counts < 50 cells/μL [74]. In one study, the case fatality rates of mpox disease were 9.4% and 20.8% overall and in HIV-positive cases, respectively, demonstrating that HIV infection is associated with a higher risk of contracting and dying from mpox [67]. In particular, PLWH with a CD4 T cell count < 50 cells/μL face a high risk of mortality due to mpox [72]. Furthermore, a cohort of PLWH and mpox, which predominantly included individuals with advanced or uncontrolled HIV infection, showed that this population was significantly more likely to experience severe mpox manifestations and prolonged disease compared with those without HIV [69]. On the contrary, other studies showed that there was no significant difference in disease severity and presentation between groups with and without HIV, especially for those with well-controlled HIV infection, suggesting that HIV status itself may not be a risk factor for mpox severity, but an indicator for increased sexual risk behaviors and, therefore, mpox transmission [75,76]. Healthcare providers and public health stakeholders should address the vulnerabilities of PLWH and prioritize early detection, prompt treatment initiation and comprehensive care in order to improve the outcomes of mpox disease in this population.

## 5. Clinical Presentation

**Figure 2:**
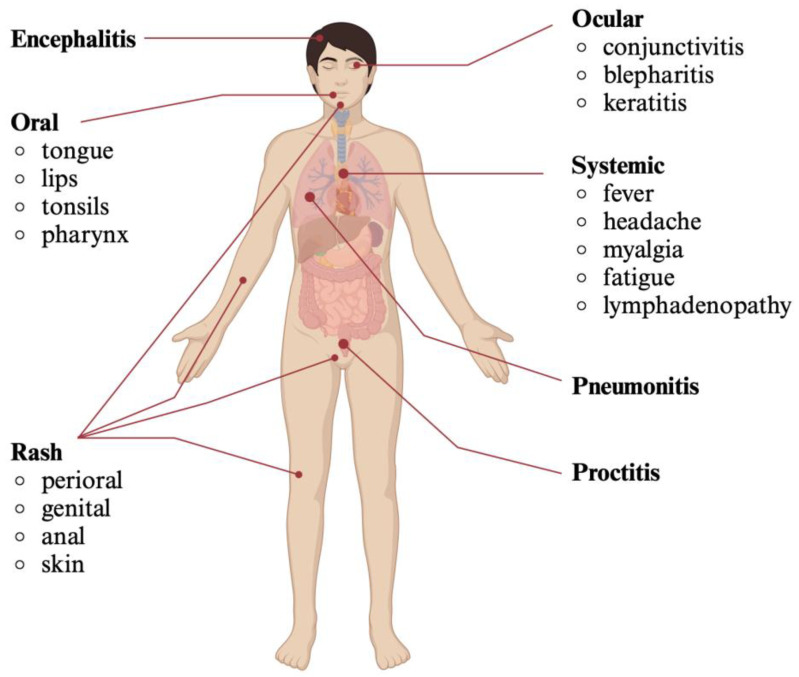
Manifestations of mpox (created using https://www.biorender.com (accessed on 7 July 2024)).

The incubation period of mpox exhibits great variability depending on the outbreak and the presumed mode of exposure [77]. It is estimated that the average duration between infection and symptom onset ranges from 5 to 13 days [8,78,79]. The clinical presentation of mpox includes both a systemic illness and system-specific symptoms (Figure 2) [58]. Studies of more recent outbreaks reveal a greater variability in the relationship between systemic disease and organ-specific symptoms [77,80]. In this review, we delineated the most common clinical presentations observed in the 2022 outbreak, categorized by organ system and with respect to the natural course of the disease.

**Figure 3:**
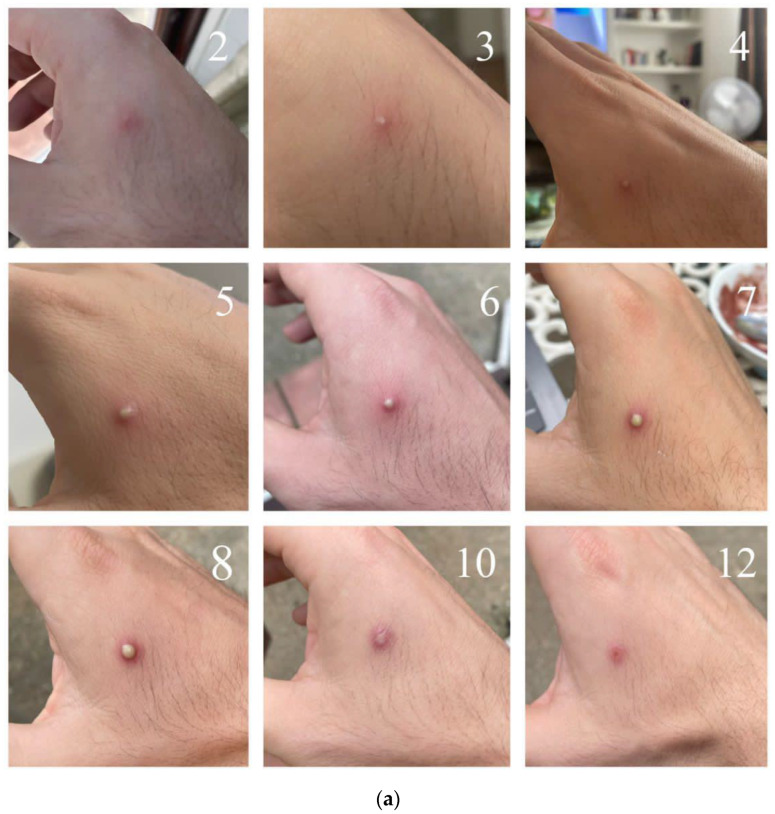

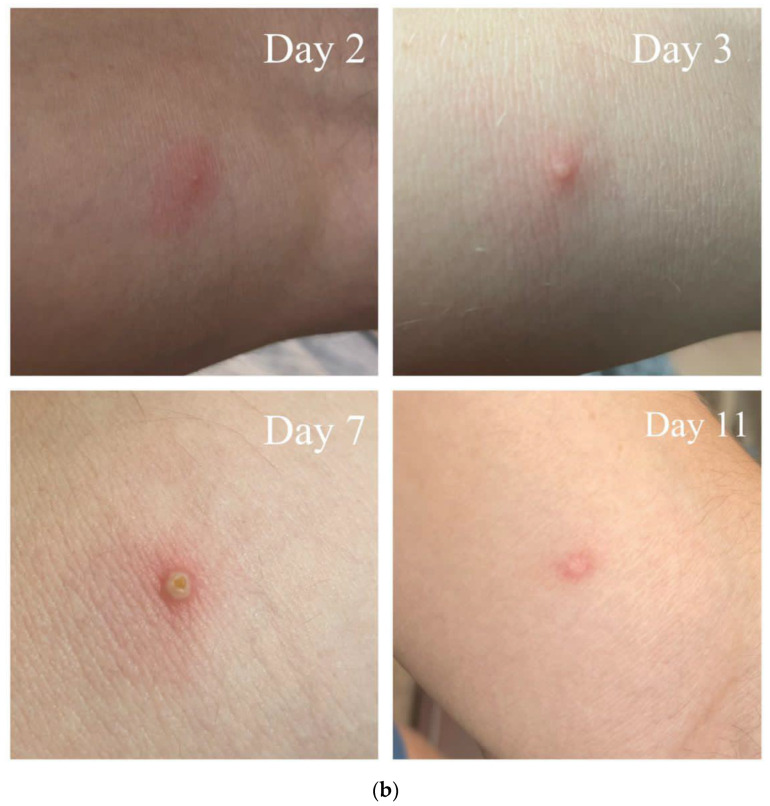
(**a**) Temporal evolution of the characteristic rash in a patient with mpox. Typical evolution of a cutaneous mpox lesion (from left to right): well-demarcated papule on an erythematous base; vesicle, pustular umbilicated lesion on an erythematous base; ulcerated lesion; crusted lesion. (**b**) Temporal evolution of the characteristic rash of mpox.

Systemic symptoms of mpox correspond with the active replication of the virus and may predate or follow the appearance of the characteristic rash [78,81]. These symptoms include, but are not limited to, fever, headache, myalgia, fatigue and lymphadenopathy, lasting for one to five days [77,78]. During the 2022 outbreak, the clinical presentation was frequently associated with rash development in the absence of systemic illness, while lymphadenopathy was usually related to the appearance of the skin rash [77]. The characteristic rash is typically well circumscribed and follows a predictable evolution timeline [77,82,83,84]. Lesions described in the 2022 outbreak typically evolved in an asynchronous manner over 7 to 14 days, starting as macules of 2 to 5 mm on an edematous background and then evolving to papules, vesicles and umbilicated pustules before crusting and healing with complete re-epithelialization (Figure 3) [78,82]. Pruritus is commonly associated with the crusting period. Rarely, lesions coalesce into plaques or ulcerate and become necrotic [78,82]. The number of lesions can vary from 1 to 20 on average, with some cases presenting up to 100 lesions [78,82].

In the recent outbreak, these lesions were more often located in the perioral, genital and perianal area, with fewer lesions appearing on the trunk or extremities, suggesting a possible correlation between sites of inoculation and lesion appearance [78,82,85]. Perioral and oral lesions are usually circular and white, commonly ulcerative in nature and with a central depression [78,82,85,86]. They may appear on the tongue, lip mucosa, tonsils or pharynx and are associated with pain and dysphagia [78,82,85,86]. Genital lesions are usually solitary, located on the prepuce or the glans of the penis in males and on the labia in females and are almost universally associated with severe pain and edema [64,78,82,85,87]. Typically, perianal and anal lesions mimic proctitis, causing painful bowel movements; they are associated with discharge and bleeding and, in rare cases, with bowel perforation [64,78,82]. Their morphology is more ambiguous, spanning from vesicles to pustules to ulcers, often resembling herpes, syphilis, lymphogranuloma venereum or other pathologies [64,78,82].

The most common ocular manifestations of mpox include conjunctivitis, blepharitis, keratitis and secondary cellulitis, leading, in some cases, to loss of vision [88,89]. The involvement of the central nervous system (CNS) in the form of encephalitis and encephalomyelitis have been described, although it has not been elucidated if these manifestations represent a consequence of mpox infection or a secondary immune-mediated phenomenon [2,90,91]. Other systems can also be affected, causing severe complications such as sepsis, necrotizing lymphadenopathy, bronchopneumonia, myocarditis, abscess formation and hemophagocytic lymphohistiocytosis [64,82,92,93,94]. In a nationwide UK cohort, severe pain and secondary bacterial infection were the most common complications in hospitalized patients with mpox [95].

## 6. Diagnosis

Mpox should be suspected in all patients with consistent clinical, laboratory and epidemiological findings [96]. It should be considered in the differential diagnosis of all patients presenting with a morphologically suspicious rash, especially if consistent symptoms from other systems are present [96,97]. A history of contact with suspected or confirmed cases or recent travel to areas where mpox is endemic or where outbreaks have been reported should be taken into account [96,97].

In terms of laboratory testing, a polymerase chain reaction (PCR) performed on material collected from typical lesions is considered to be the gold standard for mpox diagnosis. In the setting of numerous lesions, the sampling of multiple sites may increase the diagnostic accuracy [97,98]. A PCR using throat swabs and blood adds little to the diagnosis in a clinical setting [97,98]. Antibody testing for mpox may be performed when a PCR is not readily available, with a relatively narrow seroconversion window after the appearance of symptoms [99]. Despite its value in research, electron microscopy is not considered feasible for routine diagnosis [100,101]. Histopathological findings in tissues presenting typical lesions are non-specific and have little value in clinical practice [101]. A high index of suspicion and the availability of PCR testing are the cornerstones for an accurate and timely diagnosis of mpox.

Mpox needs to be differentiated from other exanthematous diseases such as chickenpox, measles, scabies, hand–mouth–foot disease, genital herpes, syphilis and other sexually transmissible infections (STIs) [102,103]. Lymphadenopathy is common in mpox, but mostly absent in chickenpox. Herpes often causes oral or anogenital polycyclic lesions, usually in people with similar previous episodes. When genital ulcers or proctitis are present, the recommended method for accurate diagnosis is PCR testing [104]. Furthermore, when sexual transmission is suspected, testing for other STIs should also be conducted [105].

## 7. Treatment

The management of mpox infection should take into account the disease severity, clinical manifestations and involvement of specific anatomic sites as well as the immunological status of the patient, the risk of progression to severe disease and the development of complications [47,106,107]. In the majority of immunocompetent patients, mpox presents as a mild clinical syndrome that can be optimally managed by supportive measures and minimal, if any, medical intervention [106]. Supportive measures should aim to optimize nutrition and hydration, pain management and the prevention of possible complications [107]. Hospitalization is usually indicated for patients experiencing severe infection who are at risk of malnourishment or dehydration due to lesions located at the level of the rectum or the oropharynx and for patients who develop complications or need intensive pain management [47].

Antiviral therapy is not indicated for all patients, but it is recommended for those with severe disease presenting with numerous confluent lesions, neurological manifestations and respiratory or multisystemic involvement [108]. Patients with a documented history of autoinflammatory and/or exfoliative skin disorders and ocular manifestations as well as patients with multimorbidity are also candidates for antiviral therapy. An immunocompromised status such as advanced HIV infection, hematological malignancy, bone marrow or solid organ transplantation, chemotherapy or radiation as well as documented autoimmune disease are all strong indicators for antiviral treatment [47,107,108]. Very young (<8 years old) and very old individuals as well as pregnant and breastfeeding people can also benefit from antiviral therapy [109,110,111]. The timing of antiviral therapy initiation is based on limited clinical data; however, early treatment initiation after the appearance of symptoms is thought to be associated with a more rapid clinical response and favorable clinical outcomes [112].

The mainstay of antiviral therapy for mpox is tecovirimat, an agent with a favorable safety profile and mild side effects whose efficacy is being increasingly supported by observational data, especially for patients with severe disease and when started early in the course of disease [77,113,114,115]. Evidence from randomized clinical trials (RCTs) is currently lacking; however, several RCTs are now ongoing. The preliminary results from PALM 007 (NCT05559099) suggest that tecovirimat is safe, but it did not improve Clade I mpox outcomes [116]. Whether or not this is a clade-specific outcome remains to be investigated. Tecovirimat is an inhibitor of the Orthopoxvirus VP37 envelope-wrapping protein, effectively limiting the formation of the extracellular virus particle, necessary for viral dissemination within the host [117,118]. A low barrier to the virus developing resistance to tecovirimat should be considered before treatment initiation and its overuse, especially in mild cases, should be avoided [47,107,119,120,121]. It is available in oral and intravenous formulations. Orally, the administered dose is 600 mg q12hr for those < 120 kg and q8hr for those > 120 kg. The intravenous form is administered at 200 mg q12hr for those < 120 kg and at 300 mg for those > 120 kg [119,120,121]. A switch from intravenous to oral treatment should be considered for patients who are able to take oral medication. Treatment typically lasts 14 days but, in selected cases, the treatment duration can be extended. Possible drug interactions with immunosuppressive agents and antiretroviral treatment should be considered before treatment initiation [119,120,121].

When tecovirimat is not available, cidofovir and brincidofovir represent alternative regimens that have been used in clinical practice with less favorable side-effect profiles and very limited data concerning efficacy [122,123,124,125,126,127]. In patients with ocular mpox, tecovirimat is strongly advised with the addition of trifluridine in the form of eye drops or ointment applied q4hr for 7 to 10 days [128]. Skin lesions that fail to heal, extend despite treatment or show changes in terms of the morphology, associated erythema or other signs of inflammation must be evaluated for bacterial superinfection and appropriate antibiotic treatment initiated [129].

## 8. Prevention

### 8.1. Contact Precautions

The implementation of effective infection control measures such as contact precautions is essential for the prevention of mpox transmission and spread within healthcare and community settings [60]. However, increased community awareness and wider surveillance of cases through publicity and education about the risk factors, prevention and treatment of mpox are crucial to control and eliminate infection as well as to minimize stigmatization [60]. In addition, the WHO and CDC guidelines highlight the importance of early detection, the isolation of cases, the appropriate use of personal protective equipment (PPE) and communication with healthcare workers and the public [130,131]. According to these guidelines, the contact precautions for mpox include the isolation of suspected or confirmed cases, the use of PPE and the implementation of strict hand-hygiene practices to minimize the risk of transmission [130]. More specifically, contact and droplet precautions should be implemented for any suspected or confirmed patient with mpox as well as airborne precautions if aerosol-generating procedures are performed [130]. Furthermore, surfaces frequently used by the patient or where patient-care activities occur as well as patient-care equipment should be cleaned and disinfected, while fabric items such as linens, hospital gowns, towels and clothes should be carefully handled and collected [130]. Also, all bodily fluids and solid waste of patients with mpox should be treated as infectious waste [130]. All patients with mpox should be advised to abstain from sex until all skin lesions have crusted, the scabs have fallen off and a fresh layer of skin has formed underneath. Condoms should be consistently used during sexual activity (receptive and insertive oral, anal or vaginal) for 12 weeks after recovery [130]. Isolation of the suspected or confirmed mpox cases, ideally in a room with negative pressure and social distancing, is needed to prevent transmission [60]. Additionally, it is important to avoid any direct contact with animals such as rodents and primates (as they can be potential virus reservoirs) as well as exposure to blood and inadequately cooked meat [60]. The quarantine of imported animals for at least 6 weeks should be implemented and laboratory research with live MPXV should be strictly regulated to minimize spill-over events [60].

### 8.2. Vaccination

Vaccination remains one of the primary strategies for the prevention and control of mpox outbreaks [82]. Vaccinia-poxvirus-based vaccines were used throughout the 20th century to eradicate human smallpox epidemics. These vaccines provide cross-protective immunity against mpox, providing a protection of about 85%. This has led to the incorporation of vaccinia-virus-based vaccines in the control efforts for outbreaks [106]. Vaccine-induced immunity lasts for a long period of time as people vaccinated against smallpox > 25 years ago are still protected against mpox, while the incidence of mpox infections significantly increased 40 years after the discontinuation of smallpox vaccination campaigns [132]. ACAM2000 is a second-generation live-attenuated vaccinia-based vaccine that was approved by the FDA in August 2007 for individuals > 18 years of age to replace the older vaccinia-virus-based vaccines and it was used for mpox prevention [133]. This vaccine is indicated for immunocompetent people and non-pregnant or non-breastfeeding people who are at a high risk of exposure [133]. It is administered once by the percutaneous route (scarification) using a bifurcated needle; a pustular-like skin reaction occurs at the injection site, forming a scab after 2–3 weeks [133]. The ACAM2000 vaccine is effective against mpox infection; however, it can cause severe adverse effects such as myopericarditis, progressive vaccinia, eczema vaccinatum, post-vaccination encephalitis and severe fetal smallpox through vertical transmission [106,134].

A modified vaccinia Ankara vaccine, also known as Jynneos in the USA, Imvamune in Canada or Imvanex in Europe, originated from the genetic modification of the vaccinia Ankara–Bavarian Nordic (MVA-BN strain) virus grown in chicken embryo fibroblasts [135]. It is a third-generation, live-attenuated, non-replicating viral vector vaccine that was approved by the FDA in September 2019 and is indicated for the prevention of human smallpox and mpox in people over 18 years of age at a high risk of these infections. It is subcutaneously or intradermally administered in two doses at least 28 days apart [135]. High immunogenicity and an excellent safety profile have been demonstrated in randomized clinical trials [136,137]. Since the 2022 outbreak, data on the effectiveness of a single-dose MVA-BN show a moderate effect in preventing mpox infection [138]. Another third-generation live-attenuated replicating vaccine is LC16m8, which was derived from the Lister (Elstree) strain of vaccinia and was licensed in Japan for immunization against smallpox and mpox [139,140]. Studies have shown that LC16m8 is highly immunogenic against mpox, with a favorable safety profile [139,140].

According to the WHO recommendations for vaccination strategies against MPXV infections, a primary preventive pre-exposure vaccination with third-generation non-replicating vaccines is only recommended for individuals at a high risk of exposure such as gay, bisexual or other MSM with multiple sexual partners as well as sex workers, healthcare workers at a high risk of exposure, laboratory personnel working directly with any poxviruses and personnel designated by the health authorities who are directly involved in the treatment of and contact with possible mpox cases [141]. Mass vaccination is not currently required nor recommended for mpox [141]. Post-exposure preventive vaccination with a third-generation vaccine is recommended in the first 3–4 days of exposure and up to 14 days for the close contacts of infected people with a high risk of exposure, but if administered between days 4 to 14 post-exposure, the vaccine only minimizes the symptoms and does not prevent infection [141]. It is worth noting that in immunocompetent individuals, the ACAM2000 vaccine can be also administered for post-exposure prevention [141]. Thus, vaccination against mpox may be an effective measure for the prevention or attenuation of the disease, controlling the epidemics of mpox along with other preventive measures such as contact precautions.

## 9. Reinfection and Post-Vaccination Infection

Although the duration of immunity provided by vaccination or a past infection is under investigation, an increasing number of case reports of possible mpox reinfections have been published since January 2023 [142,143]. For some of the possible reinfection cases, there is still a debate as to whether they are relapses of previous infections or not because confirmation from phylogenetic sequencing has not been possible. Nevertheless, there is at least one case of reinfection that has been confirmed by whole-genome sequencing [144]. The epidemiologic characteristics and risk factors for reinfection and post-vaccination infection show consistency with those described in the literature for primary mpox infections in the 2022 epidemic. Sexually active MSM (either PLWH or on PrEP) reporting multiple sexual partners and condomless sexual intercourse compose the majority of these cases [145,146]. Of note, a significant proportion of the individuals diagnosed with reinfection or post-vaccination mpox infection had a concomitant STI.

Clinical manifestations of repeated or post-vaccination infections seem to differ from those reported in the 2022 outbreak in terms of the extent and severity. To date, no deaths have been reported among these cases, while the number of individuals who required hospitalization is limited [145,147,148]. It is worth mentioning that more than 10% of the individuals diagnosed during the 2022 epidemic required hospitalization [47,64,143]. In a global case series of individuals with repeated mpox infection (mostly PLWH or on PrEP), significantly fewer lesions were observed during reinfection compared with the initial infection and the time required for the resolution of symptoms was shorter. Moreover, the extent of body involvement, the number of mucosal areas affected and analgesia requirement were lower during reinfection and none of the cases required hospitalization [145]. When compared with repeat infections, individuals with infection after vaccination showed a less extensive involvement of extragenital areas and less analgesia requirement.

In conclusion, the ever-increasing number of reinfection and breakthrough mpox cases underscores the importance of including mpox in the differential diagnosis of individuals with presumed immunity through recent infection or vaccination presenting with symptoms compatible with mpox.

## 10. Global and Community Responses

After the recognition of the first mpox cases in May 2022 in several non-endemic countries and the widespread epidemic that followed, a PHEIC was rapidly declared on 23 July 2022 [45]. To contain the spread of the disease, a rapid global response was warranted, capitalizing on the knowledge gained during the SARS-CoV-2 pandemic. The significance of international collaboration in terms of surveillance and data-sharing was immediately acknowledged. From the very beginning of the epidemic, information was collected and communicated in real time by the appropriate authorities/organizations, both at national and international levels. This led to the early identification of MSM as the population most affected as well as the risk factors associated with this epidemic. As a result, sexual health clinics were on the frontline of this epidemic. The primary goal was the early detection of mpox cases so that timely isolation and contact tracing could take place. These interventions were necessary to reduce the spread of the virus.

Due to the possible stigmatizing features of the disease and given the lessons learned from the HIV epidemic, community involvement was deemed essential to cultivate trust, establish routes of communication and reach marginalized groups [149,150]. Several successful examples of community-led initiatives have been reported. Early in the mpox outbreak in 2022, HIV/AIDS organizations in Germany and transgender women’s groups in Peru managed to reduce spread by raising awareness in the LGBTQI+ community, underlying the importance of community involvement [150]. Community engagement proved to be important in reducing stress and mpox-related stigma as well as distinguishing between real information and misinformation and conspiracy theories across social networks during the epidemic [59,151]. Moreover, at the beginning of this outbreak, a lack of trust in health systems among a significant proportion of the public after the experience of SARS-CoV-2 was still fresh. The cooperation of the state with community organizations managed to shift the sexual behavior of the population at risk to safer sexual practices, which ultimately contributed to limiting the epidemic [152,153,154]. It seems that harm-reduction strategies and sex-positive messages were much more effective than the initial guidelines, which suggested strict abstinence [152,155].

The final important pillar of the epidemic containment was the broad dispensing of available vaccines. Pre- and post-exposure vaccination programs have played a key role in the strategies against mpox in North America and Europe. Unfortunately, the global response once again fell short of what was needed. Early in the 2022 mpox global outbreak, access to vaccines was unequal across high- and low-income countries. The availability of vaccines in the African countries where the virus had been endemic for decades was negligible. On the other hand, high-income countries secured large supplies of mpox vaccines [155]. In addition, as per August 2024, only two African countries had granted Emergency Use Authorization for the MVA-BN vaccine. As a result, during the current 2023–2024 outbreak originating in DRC, the population of the affected regions was not vaccinated, favoring the rapid spread of the virus. This led international stakeholders to take measures, including the procurement and donation of hundreds of thousands of vaccine doses to affected countries [156,157]. In order to facilitate timely and increased access to vaccines, the WHO proceeded with the prequalification approval of the MVA-BN vaccine in September 2024 [158].

## 11. Challenges and Future Perspectives

While the WHO declared the end of the PHEIC for mpox in May 2023, sustained transmission of the Clade IIb virus has been established in humans, so the risk of resurgence remains a real public health threat, especially considering the current outbreak in African countries. Moreover, clinicians should be aware that previous infection and vaccination do not preclude mpox and they should investigate further when clinical suspicion is high. Data support the importance of vaccination and the recommendation that people who are at a high risk of MPXV infection should be prioritized to receive the mpox vaccine. This approach requires global vaccine availability in the areas historically affected by mpox and also in newly affected countries where access is limited. In the context of vaccine shortages when the number of those at risk exceeds vaccine availability, dose-sparing strategies or administration of at least a single dose of the vaccine to provide more widespread population coverage should be considered [138,159,160]. The protection of immunosuppressed people, including people with advanced HIV disease, through vaccination also represents a highly relevant area for research and for policymakers. Immune responses and immunity against Clade IIb MPXV are areas of active research, while phylogenetic and epidemiological analyses as well as randomized clinical trials are urgently needed.

**Figure 4:**
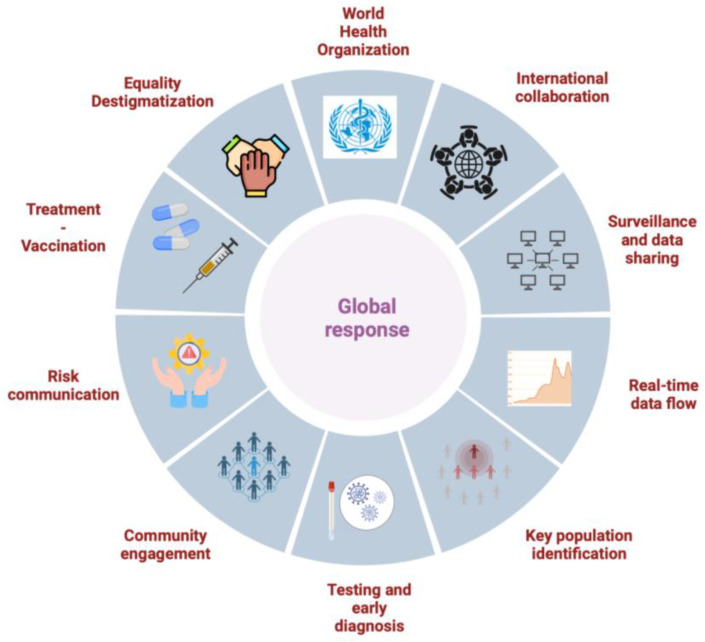
Global response components to effectively battle the current mpox outbreak and future epidemics from emerging pathogens (created using https://www.biorender.com (accessed on 12 July 2024)).

There is also a need to bolster public health surveillance so that relevant public health agencies are notified of cases of suspected relapse, reinfection and breakthrough infection to further inform policies and guidelines. For this to be accomplished, a unified, country-driven strategy with decentralized laboratories properly equipped to enhance their sequencing capacity should be adopted. The development of point-of-care tests would enable better, large-scale testing within a reasonable timeframe. The adaptation of an integrated, individualized and evidence-based management of cases, with a focus on the research and development of new treatment strategies and options, can lead to better prognosis and reduced morbidity and mortality. Finally, future public health interventions and campaigns should engage key populations and communities to ensure greater acceptance and credibility of implemented policies [161]. Focusing on providing community-led structures with the authority and capacity to act is a proven strategy that can reduce costs and optimize efficacy (Figure 4).

## Data Availability

The data underlying this article will be shared upon reasonable request to the corresponding author.
